# Dopamine signals for reward value and risk: basic and recent data

**DOI:** 10.1186/1744-9081-6-24

**Published:** 2010-04-23

**Authors:** Wolfram Schultz

**Affiliations:** 1 Department of Physiology, Development and Neuroscience, University of Cambridge, Downing Street, Cambridge CB2 3DY, UK

## Abstract

**Background:**

Previous lesion, electrical self-stimulation and drug addiction studies suggest that the midbrain dopamine systems are parts of the reward system of the brain. This review provides an updated overview about the basic signals of dopamine neurons to environmental stimuli.

**Methods:**

The described experiments used standard behavioral and neurophysiological methods to record the activity of single dopamine neurons in awake monkeys during specific behavioral tasks.

**Results:**

Dopamine neurons show phasic activations to external stimuli. The signal reflects reward, physical salience, risk and punishment, in descending order of fractions of responding neurons. Expected reward value is a key decision variable for economic choices. The reward response codes reward value, probability and their summed product, expected value. The neurons code reward value as it differs from prediction, thus fulfilling the basic requirement for a bidirectional prediction error teaching signal postulated by learning theory. This response is scaled in units of standard deviation. By contrast, relatively few dopamine neurons show the phasic activation following punishers and conditioned aversive stimuli, suggesting a lack of relationship of the reward response to general attention and arousal. Large proportions of dopamine neurons are also activated by intense, physically salient stimuli. This response is enhanced when the stimuli are novel; it appears to be distinct from the reward value signal. Dopamine neurons show also unspecific activations to non-rewarding stimuli that are possibly due to generalization by similar stimuli and pseudoconditioning by primary rewards. These activations are shorter than reward responses and are often followed by depression of activity. A separate, slower dopamine signal informs about risk, another important decision variable. The prediction error response occurs only with reward; it is scaled by the risk of predicted reward.

**Conclusions:**

Neurophysiological studies reveal phasic dopamine signals that transmit information related predominantly but not exclusively to reward. Although not being entirely homogeneous, the dopamine signal is more restricted and stereotyped than neuronal activity in most other brain structures involved in goal directed behavior.

## Background

Results from lesion and psychopharmacological studies suggest a wide range of behavioral functions for midbrain dopamine systems. The key question is, which of these many functions are actively encoded by a phasic dopamine signal compatible with rapid neuronal mechanisms? Good hints come from drug addiction and electrical self-stimulation, suggesting that dopamine activity has rewarding and approach generating effects [1,2].

We can define rewards as objects or events that generate approach and consummatory behavior, produce learning of such behavior, represent positive outcomes of economic decisions and engage positive emotions and hedonic feelings. Rewards are crucial for individal and gene survival and support elementary processes such as drinking, eating and reproduction. This behavioral definition attributes reward function also to certain nonalimentary and nonsexual entities, including money, technical artefacts, aesthetic stimulus attributes and mental events. Rewards engage agents in such diverse behaviors as foraging and trading on stock markets.

### Basic concepts

Rewards have specific magnitudes and occur with specific probabilities. Agents aim to optimize choices between options whose values are determined by the kind of the choice object and its magnitude and probability [3]. Therefore rewards can be adequately described by probability distributions of reward values. In an ideal world these distributions follow a Gaussian function, with extreme rewards occurring less frequently than intermediate outcomes. Experimental tests often use binary probability distributions with equiprobable values (each reward value occurring at p = 0.5). Gaussian and binary probability distributions are fully described by the mathematical expected value (first moment of probability distribution) and the dispersions or deviations of values from the mean, namely the (expected) variance (second moment) or (expected) standard deviation (square root of variance). Variance and standard deviation are often considered as measures of risk. In behavioral economics, the term 'risk' refers to a form of uncertainty in which the probability distribution is known, whereas 'ambiguity' indicates incomplete knowledge of probabilities and is often referred to simply as 'uncertainty'. Risk refers to the chance of winning or losing, rather than the more narrow, common sense association with loss.

Predictions are of fundamental importance for making informed decision by providing advance information about the available choice options, as opposed to guesses that occur when outcomes are unknown. As reward can be quantified by probability distributions of value, reward predictions specify the expected value and (expected) variance or standard deviation of the distribution.

Evolutionary pressure favors the energy efficient processing of information. One potential solution is to store predictions about future events in higher brain centers and calculate in lower brain centers the difference between new environmental information and the stored prediction. The discrepancy between the actual event and its prediction is called an event prediction error. Keeping up with the changing environmental situation by higher brain centers would simply involve updating the predictions with the less information containing, and less energy consuming, prediction errors rather than processing the full peripheral information every time one little thing has changed [4]. In this way higher brain centers have access to the full information about the external world for perceptions, decisions and behavioral reactions at a much lower energy cost. This fundamental property of predictions leads to the observable phenomenon of learning, as defined by changes in behavior based on updated predictions.

Animal learning theory and efficient temporal difference reinforcement models postulate that outcome prediction errors are crucial for Pavlovian and operant conditioning [5,6]. Current views conceptualize Pavlovian learning as any form of acquisition of prediction that leads to altered vegetative reactions or striated muscle contractions, as long as the outcome is not conditional on the behavioral reaction. Thus, Pavlovian reward predictions convey information not only about the reward value (expected value) but also about the risk (variance) of future rewards, which constitutes an important extension of the concept proposed by Pavlov a hundred years ago. The importance of prediction errors is based on Kamin's blocking effect [7] which demonstrates that learning and extinction advance only to the extent at which a reinforcer is better or worse than predicted; learning slows progressively as the prediction approaches asymptotically the value of the reinforcer.

### Dopamine response to reward reception

**Figure 1:**
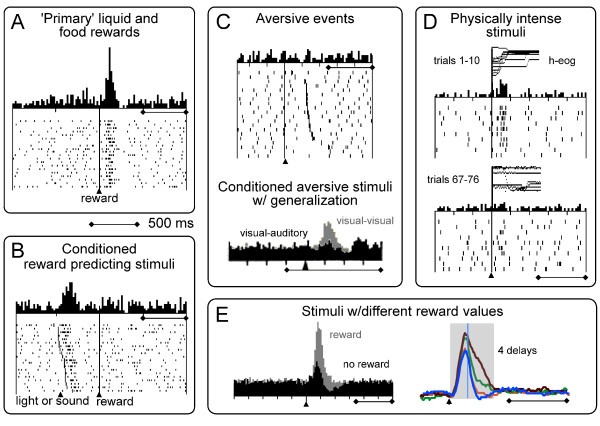
**Phasic activations of neurophysiological impulse activity of dopamine neurons**. A: Phasic activations following primary rewards. B: Phasic activations following conditioned, reward predicting stimuli. C: Top: Lack of phasic activation following primary aversive air puff. Bottom: substantial activating population response following conditioned aversive stimuli when stimulus generalization by appetitive stimuli is not ruled out; grey: population response to conditioned visual aversive stimulus when appetitive stimulus is also visual; black: lack of population response to conditioned visual aversive stimulus when appetitive stimulus is auditory. D: Phasic activations following physically intense stimuli. These activations are modulated by the novelty of the stimuli but do not occur to novelty per se. E: Left: Shorter and smaller activations followed frequently by depressions induced by unrewarded control stimuli (black) compared to responses following reward predicting stimuli (grey). Right: Activations to delay predicting stimuli show initial, poorly graded activation component (left of line) and subsequent, graded value component inversely reflecting increasing delays (curves from top to bottom). Time scale (500 ms) applies to all panels A-E. Data from previous work [29,31-33,43,59].

The majority of midbrain dopamine neurons (75-80%) show rather stereotyped, phasic activations with latencies of <100 ms and durations of <200 ms following temporally unpredicted food and liquid rewards (Figure 1A). This burst response depends on the activation and plasticity of glutamatergic NMDA and AMPA receptors located on dopamine neurons [8-12]. The burst is critical for behavioral learning of appetitive tasks such as conditioned place preference and T-maze choices for food or cocaine reward and for conditioned fear responses [9].

#### Reward prediction error coding

The dopamine response to reward delivery appears to code a prediction error; a reward that is better than predicted elicits an activation (positive prediction error), a fully predicted reward draws no response, and a reward that is worse than predicted induces a depression (negative error) [13-24]. Thus the dopamine response implements fully the crucial term of the Rescorla-Wagner learning model and resembles closely the teaching signal of efficient temporal difference reinforcement learning models [6,23].

The error response varies quantitatively with the difference between the received reward value and the expected reward value [18-23]. The prediction error response is sensitive to the time of the reward; a delayed reward induces a depression at its original time and an activation at its new time [24,25]. The quantitative error coding is evident for activations reflecting positive prediction errors. By contrast, the depression occurring with negative prediction errors shows naturally a narrower dynamic range, as neuronal activity cannot fall below zero, and appropriate quantitative assessment requires to take the full period of depression into account [26].

Thus, dopamine neurons respond to reward only to the extent to which it differs from prediction. As prediction originates from previously experienced reward, dopamine neurons are activated only when the current reward is better than the previous reward. The same reward over again will not activate dopamine neurons. If the activation of dopamine neurons has a positively reinforcing effect on behaviour, only increasing rewards will provide continuing reinforcement via dopaminergic mechanisms. This may be one reason why constant, unchanging rewards seem to lose their stimulating influence, and why we always need more reward.

#### Stringent tests for reward prediction error coding

Animal learning theory has developed formal paradigms for testing reward prediction errors. In the blocking test [7], a stimulus that is paired with a fully predicted reward cannot be learned and thus does not become a valid reward predictor. The absence of a reward following the blocked stimulus does not constitute a prediction error and does not lead to a response in dopamine neurons, even after extensive stimulus-reward pairing [27]. By contrast, the delivery of a reward after a blocked stimulus constitutes a positive prediction error and accordingly elicits a dopamine activation.

The conditioned inhibition paradigm [28] offers an additional test for prediction errors. In the task employed in our experiments, a test stimulus is presented simultaneously with an established reward predicting stimulus but no reward is given after the compound, making the test stimulus a predictor for the absence of reward. Reward omission after such a conditioned inhibitor does not constitute a negative prediction error and accordingly fails to induce a depression in dopamine neurons [29]. By contrast, delivery of a reward after the inhibitor produces a strong positive prediction error and accordingly a strong dopamine activation.

The results from these two formal tests confirm that dopamine neurons show bidirectional coding of reward prediction errors.

#### Adaptive reward prediction error coding

In a general sense, a reward predicting stimulus specifies the value of future rewards by informing about the probability distribution of reward values. Thus, the stimulus indicates the expected value (first moment) and (expected) variance (second moment) or standard deviation of the distribution.

The dopamine value prediction error response is sensitive to both the first and second moments of the predicted reward distribution at two seconds after the stimulus. In an experiment, different visual stimuli can predicted specific binary probability distributions of equiprobable reward magnitudes with different expected values and variances. As the prediction error response reflects the difference between the obtained and expected reward value, the identical magnitude of the received reward produces either an increase or decrease of dopamine activity depending on whether that reward is larger or smaller than its prediction, respectively [23]. This result suggests that value prediction error coding provides information relative to a reference or anchor value.

The dopamine coding of reward value prediction error adapts to the variance or standard deviation of the distribution. In a binary distribution of equiprobable rewards, the delivery of reward with the larger magnitude within each distribution elicits the same dopamine activation with each distribution, despite 10 fold differences between the obtained reward magnitudes (and the resulting value prediction errors) [23]. Numerical calculations reveal that the dopamine response codes the value prediction error divided by the standard deviation of the predicted distribution. This amounted to an effective normalization or scaling of the value prediction error response in terms of standard deviation, indicating how much the obtained reward value differs from the expected value in units of standard deviation. Theoretical considerations suggest that error teaching signals that are scaled by variance or standard deviation rather than mean can mediate stable learning that is resistant to the predicted risk of outcomes [30].

### Dopamine response to reward predicting stimuli

Dopamine neurons show activations ('excitations') following reward predicting visual, auditory and somatosensory stimuli (Figure 1B) [31-33]. The responses occur irrespectively of the sensory modalities and spatial positions of the stimuli, and irrespectively of the effectors being arm, mouth or eye movements. The activations increase monotonically with reward probability [18] and reward magnitude, such as liquid volume [23]. However, the dopamine responses do not distinguish between reward probability and magnitude as long as the expected value is identical [23]. Thus the activations appear to code the expected value of predicted reward probability distributions. Expected value is the more parsimonious explanation, and the noise in the neuronal responses prevents a characterization in terms of expected (subjective) utility. Note that the temporal discounting described below reveals subjective coding and might provide some light on the issue. Response magnitude increases with decreasing behavioral reaction time, indicating that the dopamine response is sensitive to the animal's motivation [19]. In choices between different reward values or delays, the dopamine responses to the presentation of choice options reflects the animal's future chosen reward [34] or the highest possible reward of two available choice options [35].

During the course of learning, the dopamine activation to the reward decreases gradually across successive learning trials, and an activation to the reward predicting stimulus develops at the same time [36,37]. The acquisition of conditioned responding is sensitive to blocking, indicating that predicton errors play a role in the acquisition of dopamine responses to conditioned stimuli [27]. The response transfer to reward predicting stimuli complies with the principal characteristics of teaching signals of efficient temporal difference reinforcement models [38]. The response shift does not involve the backpropagation of prediction errors across the stimulus-reward interval of earlier temporal difference models [27,38] but is reproduced in the original temporal difference model and in the original and more recent temporal difference implementations [6,37,39].

### Subjective reward value coding shown by temporal discounting

The objective measurement of subjective reward value by choice preferences reveals that rewards lose some of their value when they are delayed. In fact, rats, pigeons, monkeys and humans often prefer sooner smaller rewards over later larger rewards [40-42]. Thus, the subjective value of reward appears to decay with increasing time delays, even though the physical reward, and thus the objective reward value, is the same.

Psychometric measures of intertemporal behavioral choices between sooner and later rewards adjust the magnitude of the early reward until the occurrence of choice indifference, defined as the probability of choosing each option with p = 0.5. Thus, a lower early reward at choice indifference indicates a lower subjective value of the later reward. In our recent experiment on monkeys, choice indifference values for rewards delayed by 4, 8 and 16 s decreased monotonically by about 25%, 50% and 75%, respectively, compared to a reward after 2 s [43]. The decrease fit a hyperbolic discounting function.

The dopamine responses to reward predicting stimuli decreases monotonically across reward delays of 2 to 16 s [25,43], despite the same physical amount of reward being delivered after each delay. These data suggest that temporal delays affect dopamine responses to reward predicting stimuli in a similar manner as they affect subjective reward value assessed by intertemporal choices. Interestingly, the decrease of dopamine response with reward delay is indistiguishable from the response decrease with lower reward magnitude. This similarity suggests that temporal delays affect dopamine responses via changes in reward value. Thus, for dopamine neurons, delayed rewards appear as if they were smaller.

Thus, dopamine neurons seem to code the subjective rather than the physical, objective value of delayed rewards. Given that utility is a measure for the subjective rather than objective value of reward, the response decrease with temporal discounting might suggest that dopamine neurons code reward as (subjective) utility rather than as (objective) value. Further experiments might help to test utility coding more directly.

### Dopamine response to aversive stimuli

Aversive stimuli such as air puffs, hypertonic saline and electric shock induce activating ('excitatory') responses in a small proportion of dopamine neurons in awake animals (14% [33]; 18-29% [44]; 23% [45]; 11% [46]), and the majority of dopamine neurons are either depressed in their activity or not influenced by aversive events (Figure 1C top). In contrast to rewards, air puffs fail to induce bidirectional prediction error responses typical for reward; prediction only modulates aversive activations [45,46].

Aversive stimulation in anaesthetised animals produces varying but often low degrees of mostly slower, activating responses (50% [47]; 18% [48]; 17% [49]; 14% [50]) and often depressions of activity. Neurophysiological reinvestigations with better identification of dopamine neurons confirmed the overall low incidence of aversive dopamine activations in anaesthetised animals [51] and located aversively responding dopamine neurons in the ventromedial tegmental area of the midbrain [52].

Conditioned, air puff predicting stimuli in awake monkeys elicit activations in the minority of dopamine neurons, and depressions in a larger fraction of dopamine neurons (11% [33]; 13% [45]; 37% [46]). The depressant responses cancel out the few activations in averaged population responses of dopamine neurons to aversive stimuli [33] (see Figure 1C bottom, black). In one study, the conditioned aversive stimulus activated more neurons than the air puff itself (37% vs. 11% [46]), although a conditioned stimulus is less aversive than the primary aversive event it predicts, such as an air puff. The higher number of activations to the conditioned stimulus compared to the air puff suggests an inverse relationship between aversiveness and activation (the more aversive the stimulus the less frequent the activation) or an additional, non-aversive stimulus component responsible for increasing the proportion of activated neurons from 11% to 37%. Although the stimulus activations correlated positively with air puff probability in the population, they were not assessed in individual neurons [46]. A population correlation may arise from a relatively small number of positively correlated neurons within that population, and the truly aversive stimulus activations might be closer to 11% than 37%. In another study, large proportions of dopamine neurons showed phasic activations to conditioned aversive stimuli when these were presented in random alternation with reward predicting stimuli of the same sensory modality (Figure 1C bottom, grey) (65% [33]); the activations were much less frequent when the two types of conditioned stimuli had different sensory modalities (Figure 1C bottom, black) (11%). The next chapter will discuss the factors possibly underlying these unexplained activations to aversive and other, unrewarded stimuli.

Although some dopamine neurons are activated by aversive events, the largest dopamine activation is related to reward. Data obtained with other methods lead to similar conclusions. Fast scan voltammetry in behaving rats shows striatal dopamine release induced by reward and a shift to reward predicting stimuli after conditioning [53], suggesting that impulse responses of dopamine neurons lead to corresponding dopamine release from striatal varicosities. The dopamine increase lasts only a few seconds and thus has the shortest time course of all neurochemical methods, closest to electrophysiological activation. The dopamine release is differential for reward (sucrose) and fails to occur with punishment (quinine) [54]. As voltammetry assesses local averages of dopamine concentration, the absence of measurable release with quinine might hide a few activations cancelled by depressions in the dopamine population response [33]. Studies using very sensitive in vivo microdialysis detect dopamine release following aversive stimuli [55]. This response may reflect a dopamine change induced by the few neurons activated by aversive stimuli, although the time course of microdialysis measurements is about 300-500 times slower than the impulse response and might be sufficient for allowing presynaptic interactions to influence dopamine release [56]. Disruption of burst firing of dopamine neurons disrupts several appetitive learning tasks but also fear conditioning [9]. The result could suggest a learning function of aversive dopamine responses if the unspecific, generally disabling effect of lower dopamine concentration is ruled out, which remains to be shown. The specific stimulation of dopamine neurons by optogenetic methods via genetically inserted channelrhodopsin induces Pavlovian place preference conditioning in mice [57]. By contrast, a net aversive effect of dopamine stimulation would have conceivably produced place avoidance learning. These results confirm the notion of a global positive reinforcing function of dopamine systems derived from earlier lesioning, electrical self-stimulation and drug addiction work [1,2]. However, these arguments postulate neither that reward is the only function of dopamine systems nor that all reward functions involve dopamine neurons.

### Phasic dopamine activations not coding reward

Stimuli can induce alerting and attentional reactions when they are physically important (physical salience) or when they are related to reinforcers ('motivational' or 'affective' salience). Behavioral reactions to salient stimuli are graded by the physical intensity of the stimuli and the value of the reinforcer, respectively. Physical salience does not depend on reinforcement at all, and motivational salience do not depend on the valence of the reinforcers (reward and punishment).

#### Responses to physically salient stimuli

Physically intense visual and auditory stimuli induce activations in dopamine neurons (Figure 1D). These responses are enhanced by stimulus novelty [58-60] but persist at a lower level for several months provided the stimuli are sufficiently physically intense. The responses are graded according to the size of the stimuli (Figure 4 in [15]). Physical salience might also partly explain responses to primary punishers with substantial physical intensity [45]. These responses may constitute a separate type of dopamine response related to the physical salience of attention inducing environmental stimuli, or they may be related to the positively motivating and reinforcing attributes of intense and novel stimuli.

The activations to physically salient stimuli do not seem to reflect a general tendency of dopamine neurons to be activated by any attention generating event. In particular, other strong attention generating events such as reward omission, conditioned inhibitors and aversive stimuli induce predominantly depressions and rarely genuine dopamine activations [14,29]. Thus the dopamine activation by physically salient stimuli may not constitute a general alerting response. The reward response is likely to constitute a separate response that may not reflect the attention generated by the motivational salience of the reward.

#### Other non-reward coding activations

Other stimuli induce activations in dopamine neurons without apparent coding of reward value. These activations are smaller and shorter than the responses to reward predicting stimuli and are often followed by depression when the stimuli are unrewarded (Figure 1E).

Dopamine neurons show activations following control stimuli that are presented in pseudorandom alternation with rewarded stimuli [27,29,32]. The incidence of activations depends on the number of alternative, rewarded stimuli in the behavioral task; activations are frequent when three of four task stimuli are rewarded (25%-63% [27]) and become rare when only one of four task stimuli is unrewarded (1% [29]). This dependency argues against a purely sensory nature of the response.

Dopamine neurons show a rather stereotyped initial activation component to stimuli predicting rewards that occur after different delays [43]. The initial activation varies very little with reward delay, and thus does not seem to code reward value. By contrast, the subsequent response component decreases with increasing delays and thus codes (subjective) reward value (see above).

Dopamine neurons show frequent activations following conditioned aversive stimuli presented in random alternation with reward predicting stimuli; the activations disappear largely when different sensory modalities are used (65% vs. 11% of neurons [33]), suggesting coding of non-aversive stimulus components. Even when aversive and appetitive stimuli are separated into different trial blocks, dopamine neurons are considerably activated by conditioned aversive stimuli. However, the more frequent activations to the conditioned stimuli compared to the more aversive primary air puff (37% vs. 11% [46]) suggests an inverse relationship to the aversiveness of the stimuli and possibly non-aversive response components.

The reasons for these different dopamine activations might lie in generalization, pseudoconditioning or motivational stimulus salience. Generalization arises from similarities between stimuli. It might explain dopamine activations in a number of situations, namely the activations to unrewarded visual stimuli when these alternate with reward predicting visual stimuli (Figure 1E left) [27,29,32] and the initial, poorly graded activation component to reward delay predicting stimuli (Figure 1E right) [43]. Generalization might play a role when stimuli with different sensory modalities produce less dopamine activations to unrewarded stimuli than stimuli with same modalities, as seen with visual aversive and auditory appetitive stimuli (Figure 1C bottom) [33].

Pseudoconditioning may arise when a primary reinforcer sets a contextual background and provokes unspecific behavioral responses to any events within this context [61]. As dopamine neurons are very sensitive to reward, a rewarding context might induce pseudoconditioning to stimuli set in this context and hence a neuronal activation. This mechanism may underlie neuronal activations to non-rewarding stimuli occurring in a rewarding context, such as the laboratory in which an animal receives daily rewards, irrespective of the stimuli being presented in random alternation with rewarded stimuli or in separate trial blocks [46]. Pseudoconditioning may explain activations to unrewarded control stimuli [27,29,32], most activations following aversive stimuli [33,45,46] and the initial, poorly graded activation component to reward delay predicting stimuli [43]. Thus pseudoconditioning may arise from the primary reward rather than a conditioned stimulus and affect dopamine activations to both conditioned stimuli and primary reinforcers that occur in a rewarding context.

Although stimuli with substantial physical salience seem to drive dopamine neurons [15,58-60] (see above), the stimuli that induce non-reward coding dopamine activations are often small and not physically very salient. Motivational salience is by definition common to rewards and punishers and on its own might explain the activations to both reward and punishment in 10-20% of dopamine neurons. Non-reinforcing stimuli might become motivationally salient through their proximity to reward and punishment via pseudoconditioning. However, dopamine activations seem to be far more sensitive to reward than punishment. As motivational salience involves sensitivity to both reinforcers, motivational salience acquired via pseudoconditioning might not explain well the non-reward coding dopamine activations.

Taken together, many of the non-reward coding dopamine activations may be due to stimulus generalization or, in particular, pseudoconditioning. Nevertheless, there seem to remain true activations to unrewarded control stimuli and to primary and conditioned aversive stimuli in a limited proportion of dopamine neurons when these factors are ruled out. Further experiments assessing such responses should use better controls and completely eliminate all contextual reward associations with stimuli in the laboratory.

Given the occurrence of non-reward coding activations, it is reasonable to ask how an animal would distinguish rewarding from unrewarded stimuli based on a dopamine response. The very rapid, initial, pseudoconditioned and poorly discriminative response component might provide a temporal bonus for faciliating fast, default behavioural reactions that help the animal to very quickly detect a potential reward [62]. By contrast, the immediately following response component detects the true nature of the event through its graded activation with reward value [43] and its frequent depression with unrewarded and aversive stimuli [27,29,32,33] (Figure 1E). Furthermore, the dopamine system is not the only brain structure coding reward, and other neuronal systems such as the orbitofrontal cortex, striatum and amygdala may provide additional discriminatory information.

### Dopamine reward risk signal

**Figure 2:**
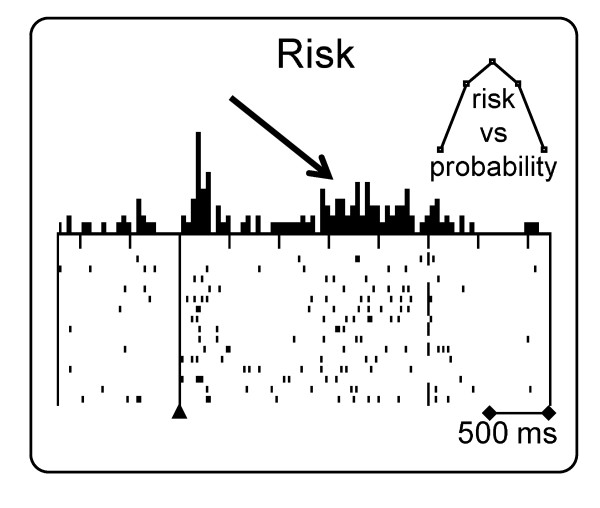
**Sustained activations related to risk**. The risk response occurs during the stimulus-reward interval (arrow) subsequently to the phasic, value related activation to the stimulus (triangle). The inset, top right, shows that risk (ordinate) varies according to an inverted U function of reward probability (abscissa) (Data from previous work [18].

If a reward signal reflects the mean reward prediction error scaled by the standard deviation of reward probability distributions, and if we view standard deviation as a measure of risk, could there be a direct neuronal signal for risk? When reward probabilities vary from 0 to 1 and the reward magnitude remains constant, the mean reward value increases monotonically with probability, whereas the amount of risk follows an inverted U function peaking at p = 0.5 (Figure 2, inset). At p = 0.5, there is exactly as much chance to obtain a reward as there is to miss a reward, whereas higher and lower probabilities than p = 0.5 make gains and losses more certain, respectively, and thus are associated with lower risk.

About one third of dopamine neurons show a relatively slow, moderate, statistically significant activation that increases gradually during the interval between the reward predicting stimulus and the reward; this response varies monotonically with risk (Figure 2) [18]. The activation occurs in individual trials and does not seem to constitute a prediction error response propagating back from reward to the reward predicting stimulus. The activation increases monotonically also with standard deviation or variance when binary distributions of different equiprobable, non-zero reward magnitudes are used. Thus, standard deviation or variance appear to be viable measures for risk as coded by dopamine neurons. Risk related activations have longer latencies (about 1 s), slower time courses and lower peaks compared to the reward value responses to stimuli and reward.

Due to its lower magnitude, the risk signal is likely to induce lower dopamine release at dopamine varicosities compared to the more phasic activations coding reward value. The relatively low dopamine concentration possibly induced by the risk signal might activate the D2 receptors which are mostly in a high affinity state but not the low affinity D1 receptors [63]. By contrast, the higher phasic reward value response might lead to more dopamine concentrations sufficient to briefly activate the D1 receptors in their mostly low affinity state. Thus the two signals might be differentiated by postsynaptic neurons on the basis of the different dopamine receptors activated. In addition, the dopamine value and risk signals together would lead to almost simultaneous activation of both D1 and D2 receptors which in many normal and clinical situations is essential for adequate dopamine dependent functions.

A dopamine risk signal may have several functions. First, it could influence the scaling of the immediately following prediction error response by standard deviation immediately after the reward [23]. Second, it could enhance the dopamine release induced by the immediately following prediction error response. Since risk induces attention, the enhancement of a potential teaching signal by risk would be compatible with the role of attention in learning according to the associability learning theories [64,65]. Third, it could provide an input to brain structures involved in the assessment of reward risk per se. Fourth, it could combine with an economic expected value signal to represent considerable information about the expected utility in risk sensitive individuals according to the mean-variance approach in financial decision theory [66]. However, the latency of about 1 s is too long for the signal to play an instantaneous role in choices under uncertainty.

## Competing interests

The author declares that he has no competing interests.

## Authors' contributions

WS wrote the paper.
